# Enhanced stability in CH_3_NH_3_PbI_3_ hybrid perovskite from mechano-chemical synthesis: structural, microstructural and optoelectronic characterization

**DOI:** 10.1038/s41598-020-68085-0

**Published:** 2020-07-08

**Authors:** Carlos A. López, Carmen Abia, Joao E. Rodrigues, Federico Serrano-Sánchez, Norbert M. Nemes, José L. Martínez, María T. Fernandez-Díaz, Neven Biškup, Consuelo Alvarez-Galván, Felix Carrascoso, Andres Castellanos-Gomez, José A. Alonso

**Affiliations:** 1 Instituto de Ciencia de Materiales de Madrid, CSIC, Cantoblanco, 28049 Madrid, Spain; 2 INTEQUI (CONICET-UNSL), and Facultad de Química, Bioquímica y Farmacia, UNSL, 5700, Almirante Brown 1455, San Luis, Argentina; 3 Institut Laue Langevin, BP 156X, 38042 Grenoble, France; 4 Departamento de Física de Materiales, Universidad Complutense de Madrid, 28040 Madrid, Spain; 5 Instituto Pluridisciplinar, Universidad Complutense de Madrid, 28040 Madrid, Spain; 6 Instituto de Catálisis y Petroleoquímica, CSIC, Cantoblanco, 28049 Madrid, Spain

**Keywords:** Chemistry, Energy science and technology, Materials science

## Abstract

Among the hybrid organic–inorganic perovskites MAPbX_3_ (MA: methyl-ammonium CH_3_–NH_3_^+^, X = halogen), the triiodide specimen (MAPbI_3_) is still the material of choice for solar energy applications. Although it is able to absorb light above its 1.6 eV bandgap, its poor stability in humid air atmosphere has been a major drawback for its use in solar cells. However, we discovered that this perovskite can be prepared by ball milling in a straightforward way, yielding specimens with a superior stability. This fact allowed us to take atomic-resolution STEM images for the first time, with sufficient quality to unveil microscopic aspects of this material. We demonstrated full Iodine content, which might be related to the enhanced stability, in a more compact PbI_6_ framework with reduced unit-cell volume. A structural investigation from neutron powder diffraction (NPD) data of an undeuterated specimen was essential to determine the configuration of the organic MA unit in the 100–298 K temperature range. A phase transition is identified, from the tetragonal structure observed at RT (space group *I*4*/mcm*) to an orthorhombic (space group *Pnma*) phase where the methyl-ammonium organic units are fully localized. Our NPD data reveal that the MA changes are gradual and start before reaching the phase transition. Optoelectronic measurements yield a photocurrent peak at an illumination wavelength of 820 nm, which is redshifted by 30 nm with respect to previously reported measurements on MAPbI_3_ perovskites synthesized by crystallization from organic solvents.

## Introduction

Hybrid perovskite solar cells (PSCs) have stepped forward at a remarkable rate, reaching over 22% energy conversion efficiency in less than a decade, and have generated a multitude of high quality works within the literature, regarding the properties of the active materials^1–7^. Their outstanding properties also have been of impact in other technological field such as photodetectors, sensors, communications, spectral analysis along with others^8–12^. These perovskites exhibit the general formula ABX_3_ (A = organic cation, typically methyl-ammonium (CH_3_NH_3_^+^): MA; B = metal, typically Pb; X = halogen). The very best overall performance has been found for methylammonium lead triiodide, CH_3_NH_3_PbI_3_ (also known as MAPbI_3_ or MAPI), exhibiting an adequate direct bandgap of ~ 1.6 eV that permits a broad absorption range over the complete visible light region, going along with a high carrier mobility and long diffusion length of charge carriers^13^. Unfortunately, its low tolerance to moisture and fast degradation upon UV light exposure and moderate temperatures have posed a chief obstacle to their commercialization^14^. Among the strategies for improving stability, tuning the composition of the perovskite, introducing hydrophobic coatings, replacing metal electrodes with carbon or transparent conducting oxides and packaging have been proposed^14–16^. Fast oxygen diffusion and iodide defects are also claimed to promote oxygen-induced degradation of perovskite solar cells: *Ab-initio* simulations indicate that iodide vacancies are the preferred sites in mediating the photo-induced formation of superoxide species from oxygen^17^.

Further investigations indeed showed that alternative compositions with partial replacement of MA by other organic molecules suitable to occupy the A positions of the perovskite, like formamidinium (FA: CH(NH_2_)_2_^+^) or even by voluminous inorganic cations like Cs^+^ or Rb^+^^6,18,19^, shifted the photoactive response to different spectral ranges; the replacement of Pb^2+^ by alternative divalent^20–22^ or aliovalent^23^ cations, or the partial or total replacement of iodide by bromide or chloride anions^24–27^ were also recently explored.

Despite the different chemical combinations and additional work in improving the chemistry, MAPbI_3_ is still the best choice in terms of performance; therefore, alternative options to utilize the potential of pristine MAPbI_3_ are worth exploring. Recently, we and other authors^28,29^ found that MAPbI_3_ specimens prepared by ball milling (mechano-chemical synthesis) exhibit a superior stability, showing no signs of degradation after several months exposed to humid air^28,29^. Ball milling is a simple, fast, cost-effective and green technology with enormous scalability potential. It has been widely applied in mineral and inorganic synthesis, but its application in organic or hybrid organic–inorganic syntheses is an underdeveloped area^30–32^, despite the obvious green credentials of such a solvent-free procedure. The phases obtained from this alternative synthesis method require a conspicuous crystal structure analysis to unveil the crystal features, which are the reason for greater stability.

The crystal structure of MAPbI_3_ has been studied several times and reported in the tetragonal symmetry at ambient temperature. Although most authors define this phase in the centrosymmetric *I*4/*mcm* space group^33–35^, some works reported it as a non-centrosymmetric material, with the *I*4*cm* space group^36^. Subsequently, second harmonic generation (SHG) measurements showed that there is no evidence for a non-centromymmetric space group^37,38^. On the other hand, the crystallographic analysis from X-ray techniques for these hybrid compounds is partial since the H atoms cannot be located. For this reason, the investigation with neutron diffraction techniques becomes essential to obtain a more detailed description of these materials, in particular concerning the distribution of the organic methyl-ammonium groups. The isotopic effect makes it advisable to study non-deuterated samples in the neutron investigation, the same ones that will be used in PSCs. Modern neutron sources allow obtaining good and exploitable neutron patterns, despite the incoherent scattering arising from natural H atoms.

It is worth mentioning here that, up to now, only a few works utilized neutron diffraction measurements to study this phase^39–42^, but none of them with samples synthesized from the mechanochemical method. Whitfield et al*.* and Harwell et al*.* used deuterated sample as powders^41,42^, while, Ren et al*.* investigated the structure in non-deuterated samples by the single-crystal method^40^. By contrast, only Weller et al*.* studied a powdered and non-deuterated phase; however, they developed a model with only two H positions, which are insufficient to build an adequate geometry for the MA cation^39^.

This work explores the reasons for the increased stability found in the ball-milled specimens, based on a detailed investigation of the crystal structure by neutron powder diffraction (NPD) techniques. We present a temperature-dependent NPD study that allowed us following the orientation of the organic MA units. This may play an important role in the properties, given the degrees of freedom for internal motion of MA groups within the PbX_6_ network. Additionally, we present atomic-resolution STEM images, which had been elusive so far given the instability of the hybrid perovskites specimens to the convergent electron beam, displaying unreported features at microscopic level. Moreover, optoelectronic measurements have been performed to evaluate the potential of this material as a PSC, showing a redshift in the light absorption, thus opening the energy range of usable visible light of this mechano-chemically synthesised, robust specimen.

## Experimental section

MAPbI_3_ was synthesized in polycrystalline form by mechano-chemical synthesis (ball milling) from stoichiometric amounts of PbI_2_ and MAI. The total mass of reactants was 1.5 g, which were weighed and mixed with 20 zirconia balls (5 mm diameter) in a N_2_-filled glove-box. The reaction took place in a Retsch PM100 mill for 4 h at 400 rpm, in a sealed zirconia-lined jar with N_2_ atmosphere. Preliminary identification was performed by laboratory X-ray diffraction (XRD); the patterns were collected on a Bruker D8 diffractometer with Cu-K_α_ (λ = 1.5418 Å) radiation; the 2θ range was 4° up to 90° with increments of 0.05°.

The NPD patterns at room temperature (RT) were collected in the D20 diffractometer, at the Institut Laue Langevin (ILL), Grenoble (France) with a take-off angle of 90° and a wavelength of 1.540 Å. The non-deuterated sample was contained in a 6 mm diameter vanadium cylinder. It was introduced in a standard “orange” cryostat and measured at 298 K (RT) for 3 h, and then cooled down to 180 K, 140 K, and 100 K, acquiring good statistics NPD patterns for 3 h each. All the patterns were analyzed with the Rietveld method using the *FullProf* program^43,44^. A pseudo-Voigt function was chosen to generate the line shape of the diffraction peaks. The background was interpolated between regions devoid of reflections. The following parameters were refined in the final run: scale factor, background coefficients, zero-point error, pseudo-Voigt corrected for asymmetry parameters, positional coordinates, anisotropic displacement factors and occupancy factors. The coherent scattering lengths for Pb, I, C, N and H were, 9.405, 5.28, 6.646, 9.36 and −3.739 fm, respectively.

The Scanning Electron Microscopy (SEM) images were obtained on a Hitachi instrument, model TM-1000, coupled to an energy-dispersive X-ray spectrometer (EDX), working with an acceleration voltage of 15 kV and 60 s of acquisition time. The sample preparation for scanning transmission electron microscopy (STEM) consisted only of the dispersion of fine powder of CH_3_NH_3_PbI_3_ sample onto the carbon grid, without using any solvent. The STEM images are performed in the JEOL ARM200 CF microscope equipped with Gatan Quantum spectrometer for electron energy loss spectroscopy (EELS). The microscope was operated at 80 kV in order to minimize the electron beam-induced damage. The images shown are taken with the high-angle annular dark field (HAADF) detectors. The random noise in EELS data is filtered out using the principal component analysis^45^.

In order to characterize the optoelectronic properties of the as-synthesized MAPbI_3_ material we placed two small drops of silver conductive paint (RS 186-3593) onto a cold-pressed pellet of material (1.5 ton), separated by 130 µm. A source measure unit (Keithley 2450) was employed to apply a bias voltage and measure the current flow between the electrodes. A fiber coupled tunable light source (a Xe lamp with a monochromator, Bentham TLS120Xe) was used to illuminate the sample in a wavelength range of 460 nm to 1,060 nm. The illumination was focused on the surface of the sample forming a circular spot of 400 µm in diameter.

## Results and discussion

**Figure 1:**
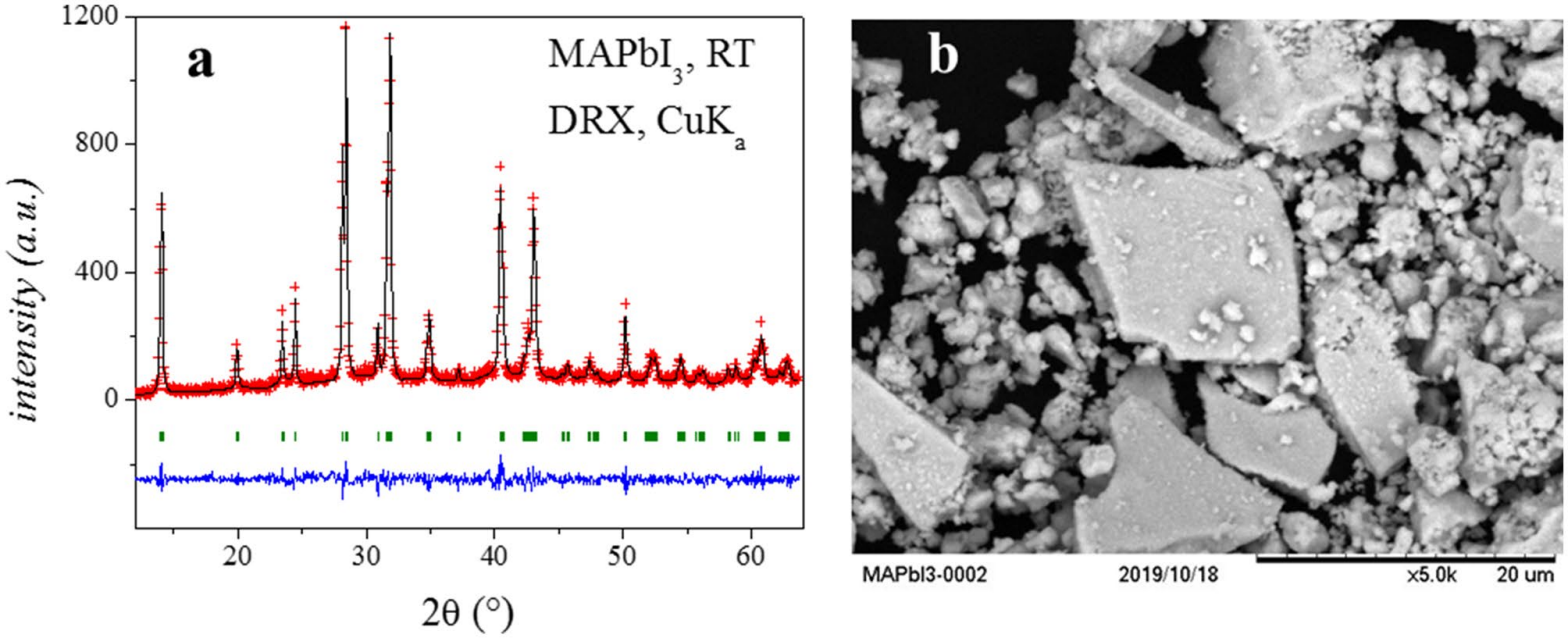
(**a**) Le-Bail refinement from a laboratory XRD pattern at RT of MAPbI_3_. (**b**) SEM image of as-prepared sample.

After the mechano-chemical process, the MAPbI_3_ sample was recovered in a N_2_-filled glove box as a well-crystallized powder, as shown on the XRD pattern illustrated in Fig. 1a. A pattern-matching procedure (Le-Bail fit) demonstrated that all the peaks can be indexed in the tetragonal *I*4*/mcm* (No. 140) space group, with unit-cell parameters *a* = *b* = 8.878(1) Å, *c* = 12.678(2) Å. No impurities or unreacted materials were observed by XRD. Figure 1b illustrates a typical SEM image of the as-prepared MAPbI_3_ perovskite. Despite the collision of high-energy ZrO_2_ balls with the reactants and products, quite large particles with sharp edges and large surfaces appear in the picture, mixed with smaller pieces with less-defined shapes. It seems that the growth of large microcrystals (with tenths of µm as maximal dimensions) is possible after 4 h of reaction, suggesting that microcrystals grow at the expense of the residual powder. The chosen time of 4 h was a compromise between an incomplete reaction and lack of crystallinity for inferior times, and a sample with excessive microestructural defects, obtained for times longer than 4 h. This justifies the good crystallinity displayed in the diffraction patterns, assessing the goodness of this method and the accurately selected conditions to obtain well-crystalized samples, comparable with those prepared with standard solution chemistry. This solvent-free procedure additionally leads to robust materials with enhanced chemical stability. The results from EDX analysis showed well-defined peaks corresponding to lead and iodine, with the determined weight % of these elements (I: 66.8 (64.8); Pb: 33.2 (35.2)) in reasonable agreement with the nominal values (in parentheses).

### Neutron diffraction investigation

The crystal structure of MAPbI_3_ was refined in the tetragonal centrosymmetric *I*4/*mcm* space group, from the neutron powder diffraction patterns collected at RT and 180 K, as previously reported^34,35,39,40^. Additional patterns collected at lower temperatures (140 and 100 K), corresponding to the orthorhombic *Pnma* symmetry, showed conspicuous differences, as described in detail below.

#### Tetragonal symmetry

In the *I*4/*mcm* space group the covalent framework PbI_6_ was defined by placing Pb at 4*c* (0,0,0) sites, I at 4*a* (0,0,1/4) and 8*h* (*x*,*x* + ½,0) Wyckoff sites. The organic cation is disordered around the 4*b* site (0,1/2,1/4) and the centre of the MA unit is displaced from the geometrical centre of this cage. In order to simulate the delocalization of this organic cation, the C and N atoms were placed at random in two positions at the 16*l* (*x*,*x* + ½,*z*) site and six H atoms in the 32* m* (*x*,*y*,*z*) site. This model is comparable to the one reported by Whitfield et al*.* and Ren et al*.*^40,41^ where the MA geometry is respected. However, there is a difference between the two reports, and it resides in the disorder end-to-end of the MA unit. An ordered case leads to a fourfold situation whereas the C/N disorder increases this MA delocalization to an eightfold case. In other words, and considering the H⋯I proximity, in the first case the H-bond interactions are only of N–H⋯I type, while in the second case the N–H⋯I and C–H⋯I interactions are equally likely. Taking into account this fact, the C/N occupation factors were refined at both temperatures (180 and 298 K). From these NPD data we observed that, at RT, the MA presents end-to-end disorder, while at 180 K this disorder is partial. Hence, the probability of N–H⋯I and C–H⋯I interactions are 50/50 and 80/20 at 298 and 180 K, respectively. Our data display an increase in the N–H⋯I interaction as temperature decreases, as it is expected in terms of electronegativity and thermal agitation.

**Figure 2:**
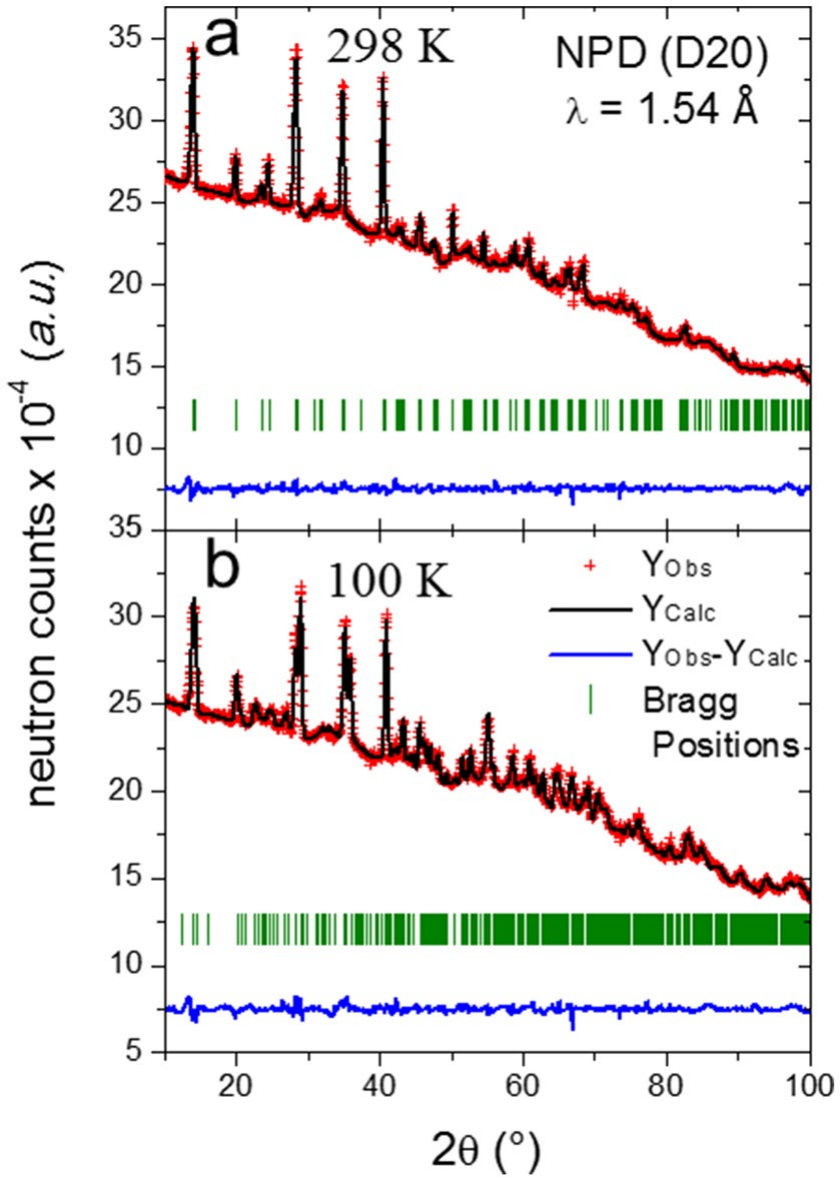
Observed (crosses) calculated (black line) and difference (blue line) profiles after the Rietveld refinement from NPD data at (**a**) 298 K and (**b**) 100 K corresponding to tetragonal (*I*4/*mcm*) and orthorhombic (*Pnma*) symmetry, respectively.

**Figure 3:**
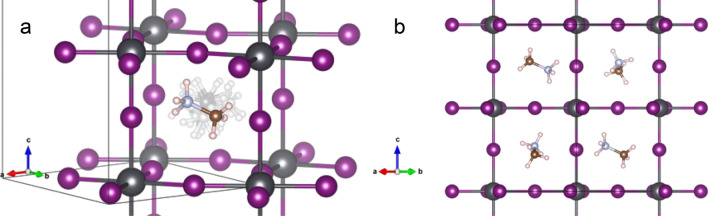
Two alternative views of the tetragonal structure observed at 298 K. (**a**) 3D view highlighting the octahedral tilting (a^0^a^0^c^−^) and the superimposed CH_3_NH_3_^+^ units, which are disordered in this temperature range (see text), (**b**) idealized projection along [110] showing four possible configurations of the CH_3_NH_3_^+^ unit in adjacent cages.

Figure 2a shows the Rietveld plot at 298 K. The main crystallographic data in the tetragonal symmetry at 298 K and 180 K are included in Supplementary Table S1 and S2, respectively, and the Rietveld plot at 180 K is displayed in Supplementary Fig. S1a. Figure 3a shows a schematic view of the tetragonal crystal structure where the configuration of the MA unit is highlighted, and Fig. 3b illustrates the fourfold possibilities of orientation of the MA units. It is worth noticing that the unit-cell volume at room temperature (991.49 (1) Å^3^) is conspicuously smaller than that reported by Ren et al*.*^40^ and Whitfield et al*.*^41^ (995.6 (2) Å^3^ and 994.97 (2) Å^3^, respectively). The present unit-cell volume also agrees with our previous report of a ball-milled sample from synchrotron X-ray diffraction (992.45 (6) Å^3^)^28^. This volume reduction also is observed at lower temperatures, as illustrated in Supplementary Fig. S2. This feature supports the greater compactness of the present sample: in defective samples, the absence of some iodide anions increases the electrostatic repulsion of Pb^2+^ around the vacant site, thus increasing the lattice size. Hence, this observed reduction can be related to a less defective sample. Moreover, the refinement of the occupancy factors for the iodide anions from NPD data gives strictly stoichiometric values (Supplementary Table S1), which could be strongly related to the stability of this material, since the mechanism of degradation seems to involve the insertion of different species at the I vacancies^17,46,47^.

#### Orthorhombic symmetry

**Figure 4:**
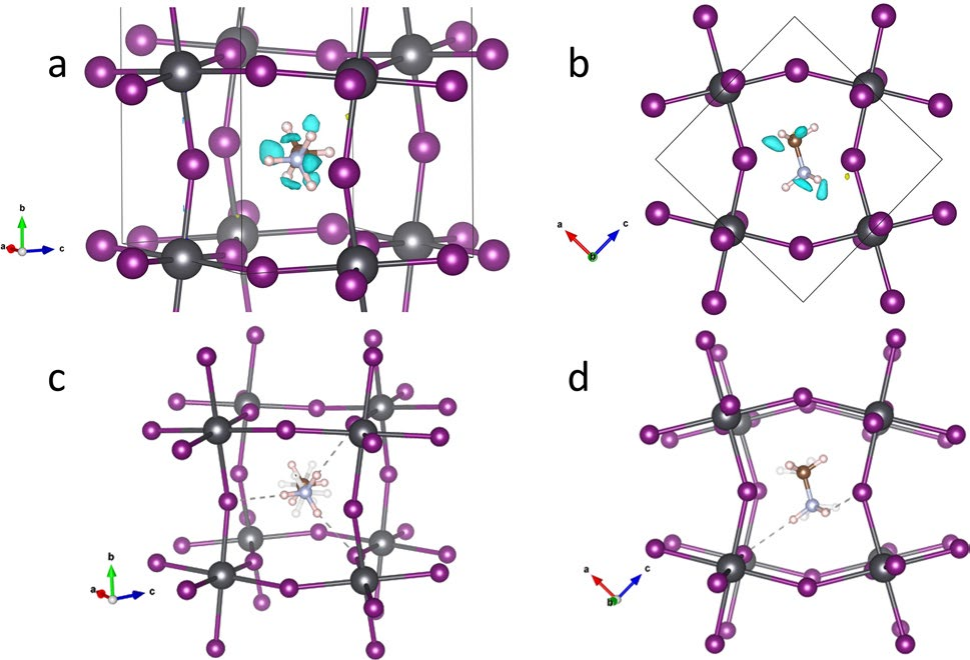
(Top) Two views of the orthorhombic crystal structure of MAPbI_3_, showing negative areas in the Difference Fourier Maps suggesting rotations of the MA molecule along the C–N axis (**a**) b-axis vertical (**b**) along b axis (bottom). Two views of the orthorhombic crystal structure of MAPbI_3_, highlighting the H-bond interactions with adjacent I atoms (**c**) approximately along [101] direction, (**d**) along b axis; note the in-phase tilting of the PbI_6_ octahedra.

The phase transition from the tetragonal to the orthorhombic phases of MAPbI_3_ has been described to happen at 160 K^41^. The 140 K and 100 K NPD patterns could thus be indexed in the orthorhombic symmetry, belonging to the *Pnma* space group. In this case, the Pb atoms are allocated in the 4*b* (0,0,1/2) site and the two types of iodide, I1 at 4*c* (*x*,1/4,*z*) and I2 at 8*d* (*x*,*y*,*z*) sites. The organic cation is around the 4*c* (1/2,1/4,1/2) position and the geometric centre of MA is displaced from this point. In the orthorhombic symmetry, the MA unit is not delocalized, and the C and N atoms were placed in 4*c* (*x*,1/4,*z*) sites and the H are distributed in both 4*c* (*x*,1/4,*z*) and 8*d* (*x*,*y*,*z*) sites. This model leads to a good starting point, which was improved from Difference Fourier Maps (DFM). The DFM displayed in Fig. 4a,b reveal the presence of non-negligible negative densities between the H atoms, which can be explained considering that the MA molecule can also be rotated by 180° along the C–N axis.

This additional position of MA was considered with four additional H atoms. The occupancy of these two sets of H atoms was refined in order to obtain the probability for each position. Such a new scenario leads to a non-negligible improvement in the refinements, mainly for the pattern collected at 140 K. The final Rietveld refinements at 100 and 140 K are plotted in Fig. 2b and Supplementary Fig. S1b. The main crystallographic data for both temperatures are listed in Supplementary Tables S3 and S4. From these data, it is possible to calculate that the contribution of the additional position of MA is 24.3% and 16.9% at 140 and 100 K, respectively. These results suggest that this additional orientation of MA is only present immediately below the transition from the tetragonal phase and its contribution quickly disappears at lower temperatures. The possible orientations of MA units are illustrated in Fig. 4c,d. The MA unit represented with solid lines corresponds to the most likely state; the H-bond interactions with I atoms are indicated with dashed lines.

#### Tetragonal-orthorhombic phase transition

The phase transitions in hybrid perovskites are well known in terms of the framework of corner-linked PbI_6_ octahedra^41,48^. In general, they behave as the conventional purely inorganic counterparts, which have been meticulously characterized for the transition metal oxides^49–51^. In many of them, the main component of these phase transitions, driven by different thermodynamic parameters, mainly temperature and pressure, can be analysed as a function of the rotation of rigid octahedra, following symmetry group-subgroup relationships^52,53^. Indeed, the two MAPbI_3_ phases identified in the 100–298 K temperature range belong to the well-known *I*4/*mcm* and *Pnma* superstructures of perovskite, characterized with the Glazer notations^54^ as *a*^0^*a*^0^*c*^−^ and *a*^−^*b*^+^*a*^−^, respectively, for the mentioned space groups. In both cases the unit-cell parameters are related to pseudo-cubic parameter as √2*a*_c_ × √2*a*_c_ × 2*a*_c_ and √2*a*_c_ × 2*a*_c_ × √2*a*_c_ for *I*4/*mcm* and *Pnma*, respectively.

**Figure 5:**
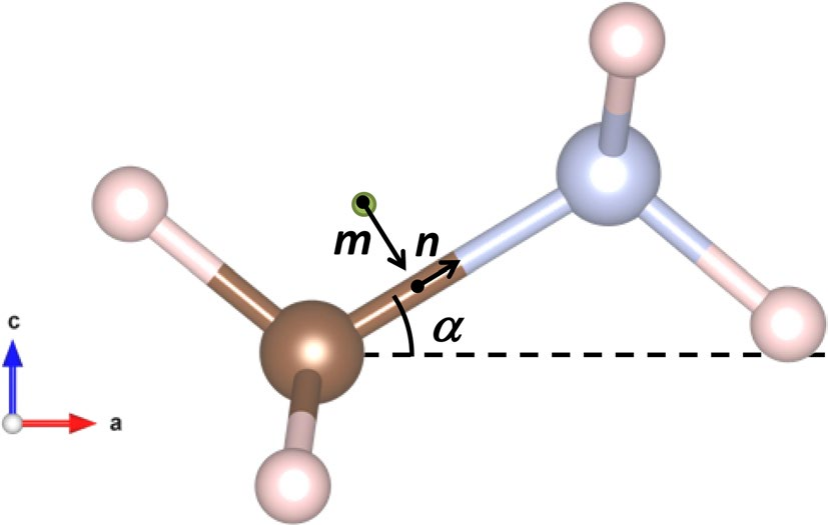
***m***, ***n*** and ***α*** parameters that define the configuration of CH_3_NH_3_^+^ organic unit. The green point corresponds to the centre of the perovskite site.

It is interesting to analyse the evolution of the MA configuration in both phases, for which three parameters were calculated: ***α*** (angle between MA and *a-b* plane or *a-c* plane for tetragonal or orthorhombic models, respectively), ***m*** (offset of MA respect to the A site of the perovskite: the ideal 4*b* (0,1/2,1/4) and 4*c* (1/2,1/4,1/2) sites for tetragonal and orthorhombic models) and ***n*** (lineal displacement of MA along C–N bond). Figure 5 illustrates a schematic view of these parameters and Table 1 lists the obtained values in addition with the disorder of C/N and the 180° rotated phase along the C–N bond. Such values reveal that the MA changes are gradual and start before reaching the phase transition. The mis-orientation and offset of the organic molecule with the crystallographic axes progressively decrease in the tetragonal phase, and disappear in the orthorhombic structure. The C/N disorder totally disappears in the *Pnma* phase, which is driven by the formation of stronger N–H⋯I hydrogen bonds with the tilted corners of the octahedra. The Pb–I–Pb angles also decrease with temperatures, implying more pronounced tilting angles, as expected.

**Table 1:** Main characteristic parameters of MA behavior at different temperatures.

	Tetragonal		Orthorhombic		
	298 K	180 K	140 K	100 K	10 K*
*α* (°)	25.2	23.9	0	0	0
*m* (Å)	0.17	0.03	0	0	0
*n* (Å)	0.23	0.33	− 0.10	− 0.11	− 0.17
C/N disorder	100%	16.2%	0%	0%	0%
180° rotated Disorder MA'	100%	100%	48.6%	33.8%	0%
Pb–I2–Pb (°)	180	180	163.59 (6)	162.49 (6)	162.55 (9)
Pb–I2–Pb (°)	163.3 (2)	156.9 (1)	151.35 (6)	150.77 (5)	150.07 (6)

*Values correspond to a deuterated sample reported by Whitfield et al*.*^41^.

The role of H⋯I interactions in the MA conformations of this hybrid material is better appreciated in Table 2, where the observed H-bond interactions at different temperatures are listed. The observed values are in agreement with the previous reports from both crystallographic and theoretical studies. The H-bond distances also follow a gradual change: the shortening of H11⋯I1 and H12⋯I1 and the lengthening of H13⋯I2 distances reveal that MA becomes gradually closer to [100] direction in the orthorhombic cell. Also, in the *Pnma* space group, the H-bond distances of the MA units rotated by 180° are longer than those of the majority form (see Table 2): this additional state of MA is probably a residual form of the delocalization present in the tetragonal symmetry.

**Table 2:** Main H-bond distances of MA at different temperatures.

	Tetragonal			Orthorhombic		
	298 K	180 K		140 K	100 K	10 K*
H11⋯I1	2.889 (5)	2.871 (7)	H11⋯I1	2.627 (4)	2.586 (3)	2.625(3)
H12⋯I1	3.059 (3)	3.012 (3)	H12⋯I2	2.723 (3)	2.728 (2)	2.696(2)
H13⋯I2	3.279 (7)	3.313 (5)	H13⋯I1^†^	3.179 (9)	3.11 (1)	–
			H14⋯I2^†^	3.565 (8)	3.507 (9)	–

*Values correspond to a deuterated sample reported by Whitfield et al*.*^41^.

^†^These distances correspond to the 180° rotated MA.

### Scanning transmission electron microscopy (STEM)

**Figure 6:**
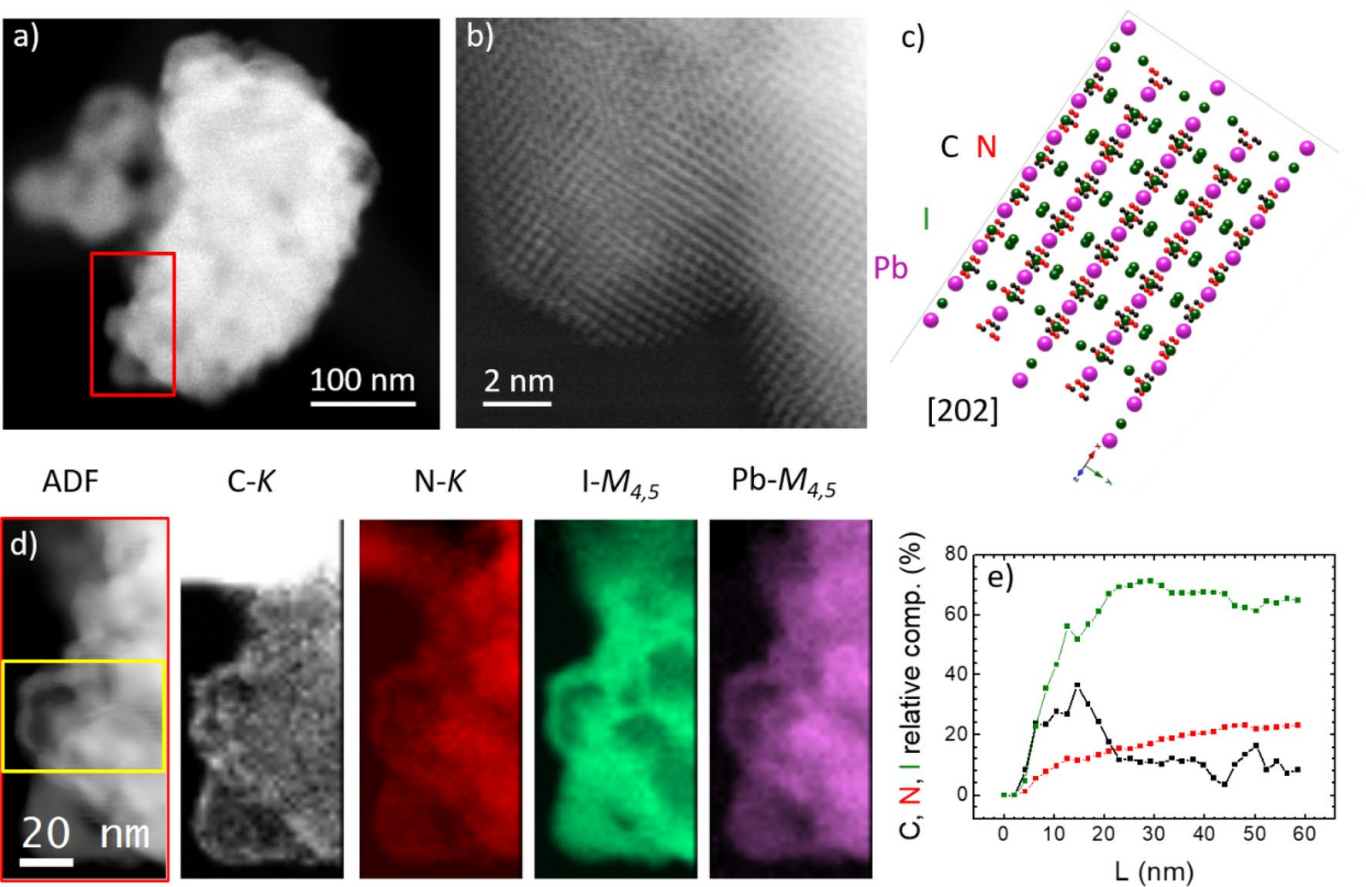
(**a**) HAADF image of one MAPbI_3_ grain. Red rectangle indicates the region where EELS data are taken. (**b**) High resolution HAADF image of a part of the grain. (**c**) Crystal in [202] zone axis based on the model taken from neutron characterization. (**d**) EELS analysis: simultaneous annular dark field image and carbon, nitrogen, iodine and lead elemental maps. (**e**) Profile in horizontal direction of chemical composition taken from the area of yellow rectangle in (**d**).

Figure 6 illustrates the electron microscopy images and elemental maps obtained by EELS spectroscopy in the submicron-size MAPbI_3_ grain. A typical grain is shown in panel a and the high-resolution HAADF image is shown in panel b. MAPbI_3_ is very sensitive to the electron beam, and a convergent electron beam, as used in atomic-resolution STEM imaging, usually destroys the microcrystal^55–60^. However, some nanocrystals endured the electron beam long enough to enable the atomic resolution imaging. Panel b shows one of these images: a MAPbI_3_ crystallite oriented approximately along the [202] tetrahedral zone axis. Only the heaviest atoms, i.e. Pb atoms can be distinguished in this image. Panel c shows the crystal model based on the crystallographic data obtained in this report in corresponding colors.

We have performed the EELS elemental analysis in order to verify the chemical composition of this grain. The elemental maps are taken from the region marked by the red rectangle in panel a. Simultaneous ADF image and maps based on C-*K*, N-*K*, I-*M*_*4,5*_ and Pb-*M*_*4,5*_ edges are shown in panel d. The grain shown in this figure lied on one of the bridges of the underlying carbon grid: this is why the upper part of the carbon elemental map shows the excess of carbon signal. We have quantified the relative composition of C, N and I using the Gatan Digital Micrograph script. Pb cannot be quantified together with lighter elements because its *M*_*4,5*_ edge is taken in a separate spectrum. The horizontal profile of these composition maps taken in the region marked by a yellow rectangle in the simultaneous ADF is shown in panel e. The relative composition data is averaged over 40 nm in vertical direction—this is the height of the yellow rectangle—in order to suppress the noise. This averaging gives a satisfactorily good data, which yield the approximate composition: 60% of iodine, 20% of nitrogen and 20% of carbon. This is the exact composition of these three elements in (CH_3_NH_3_)PbI_3_: 3:1:1. Moreover, it is particularly interesting to consider the profile of I versus N (since C is partially masked by the grid), indicating that I concentration increases much faster (with a bigger slope) when shifting into the inner regions of the crystal: I composition reaches saturation beyond 20 nm whereas N is still increasing up to 60 nm or more. This suggests that the iodide sublattice is fully stoichiometric in the bulk material, with negligible number of vacancies beyond the surface, whereas, for the organic methylammonium molecule, the existence of some bulk sub-stoichiometry is conceivable. It seems that the robustness of the crystal strongly relies on the PbI_6_ framework, and this is solidly built by mechano-chemical synthesis. Thus, the combination of chemical analysis and crystalline structure demonstrates that we have a robust MAPbI_3_ compound that is considerably more stable than the same material grown from the liquid phase^61^ and can withstand the aggressive conditions upon illumination with the electron beam.

### Optoelectronic properties

**Figure 7:**
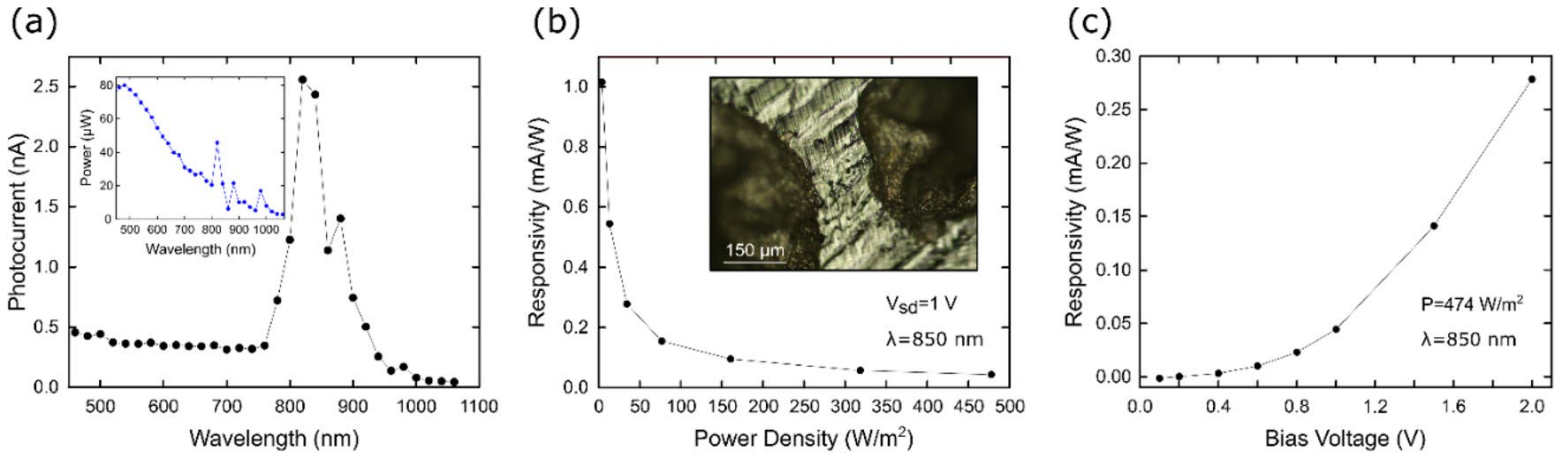
(**a**) Photocurrent of the device as a function of the light wavelength (bias voltage of 1 V). The inset displays the light power of the tunable light source as a function of the wavelength. (**b**) Responsivity as a function of the power density with a LED illumination source. The inset shows the measured pellet and the two silver paint drops that were used as electrodes. (**c**) Responsivity as a function of the bias voltage. A LED illumination source was employed to illuminate the sample.

The optoelectronic properties were directly measured in a pressed pellet of the as-grown ball-milled MAPbI_3_. No solvents were used in any stages of the process. The optoelectronic properties are comparable to those observed on MAPbI_3_ prepared by the more conventional wet-chemistry. In particular, the photocurrent values are rather similar, as discussed below. However, the peak photo-response is redshifted, indicating a reduced band-gap, which may be advantageous for photovoltaic applications. The current (at bias voltage of 1 V) was measured while the illumination is switched ON and OFF. The photocurrent was determined by subtracting the dark current from the current upon illumination (Fig. 7a). We observe a photocurrent peak of 2.5 nA at an illumination wavelength of 820 nm. Note that this value is redshifted with respect to previously reported measurements based on MAPbI_3_ material synthesized by temperature-lowering, solvent-induced crystallization or bottom seeded solution growth methods^62–64^. While the optoelectronic properties are comparable to those observed on MAPbI_3_ prepared by the more conventional wet-chemistry techniques, the peak photo-response is redshifted, indicating a reduced band-gap. This may be a result of the fully stoichiometric nature of the perovskite octahedral framework and superior compactness of the PbI_6_ framework, with respect to other materials prepared by solution chemistry. The observed shift with respect to the previously reported measurements is about 30 nm. The responsivity, a useful figure of merit to facilitate the comparison of different photodetectors, can be extracted from the photocurrent as:

1 $$R=\frac{{I}_{ph}}{P}\cdot \frac{{A}_{spot}}{{A}_{sample}}$$

where ${I}_{ph}$ is the photocurrent, $P$ is the effective power, ${A}_{spot}$ is the area of the focused spot and ${A}_{sample}$ is the illuminated active area of the device^65^.

Figure 7b and c show the responsivity as a function of the power density (bias voltage of 1 V) and as a function of the bias voltage (power density of 474 W/m^2^), respectively, using an 850 nm LED illumination source (M850F2 fiber-coupled LED from Thorlabs). This value increases at lower power densities and at higher bias voltages, as reported in previous works^64,66,67^. This effect is due to the photogating mechanism, where higher power densities emitted from the light source result in saturation of the trap states, which leads to a lower responsivity^68,69^. It is worth commenting that we decided plotting photocurrent in Fig. 7a because of the non-homogeneous power spectral density of the light source employed (we provide the illumination power distribution as an inset in Fig. 7a). In case of devices with a non-linear photocurrent vs. power dependence (this is the case, as evidenced in Fig. 7b) dividing the photocurrent by the power density reaching de device would provide an conceptually correct responsivity spectrum only if the power density is kept constant for all wavelengths. Nonetheless, the shape of the spectrum does not change sizeably (see in Supplementary Fig. S3 the Iph/power *vs* wavelength representation).

## Conclusions

We have synthesized a well-crystallized MAPbI_3_ perovskite from a mechano-chemical solvent-free ball milling method, which demonstrates to yield a robust specimen against ambient conditions. The crystallographic features were analyzed by neutron powder diffraction in the 100–298 K temperature range, where a tetragonal *I*4*/mcm* and an orthorhombic *Pnma* phases are identified, in agreement with previous reports. Neutrons permitted determining the configuration of the organic CH_3_NH_3_^+^ units within the perovskite cages, showing a progressive evolution within the stability ranges of the tetragonal and orthorhombic phases. A conspicuously smaller unit-cell volume was found in comparison with samples obtained from traditional solvent-induced crystallization, which is related to the superior compactness of the PbI_6_ framework, with a less defective nature. The robustness of the material enabled withstanding the aggressive conditions under illumination of a convergent electron beam and enabled atomic resolution imaging. EELS analysis also suggests that the iodide sublattice is fully stoichiometric in the bulk material, with negligible number of vacancies beyond the surface, whereas for the organic methyl-ammonium molecule, the existence of some bulk sub-stoichiometry is conceivable. It seems that the robustness of the crystal strongly relies on the PbI_6_ framework, and this is solidly built by mechano-chemical synthesis. Finally, the optoelectronic properties of our MAPbI_3_ specimen, implemented in a photodetector device, denoted a photocurrent peak at an illumination wavelength of 820 nm, reaching 1 mA/W of responsivity. This maximum is redshifted by 30 nm with respect to those materials synthesized by solvent-induced crystallization, which is also a result of the more compact octahedral framework.

## Supplementary information

Supplementary information
