# Author Correction: Enhanced stability in CH_3_NH_3_PbI_3_ hybrid perovskite from mechano-chemical synthesis: structural, microstructural and optoelectronic characterization

**DOI:** 10.1038/s41598-022-12534-5

**Published:** 2022-05-19

**Authors:** Carlos A. López, Carmen Abia, Joao E. Rodrigues, Federico Serrano‑Sánchez, Norbert M. Nemes, José L. Martínez, María T. Fernandez‑Díaz, Neven Biškup, Consuelo Alvarez‑Galván, Felix Carrascoso, Andres Castellanos‑Gomez, José A. Alonso

**Affiliations:** 1 Instituto de Ciencia de Materiales de Madrid, CSIC, Cantoblanco, 28049 Madrid, Spain; 2 INTEQUI (CONICET‑UNSL), and Facultad de Química, Bioquímica y Farmacia, UNSL, 5700, Almirante Brown 1455, San Luis, Argentina; 3 Institut Laue Langevin, BP 156X, 38042 Grenoble, France; 4 Departamento de Física de Materiales, Universidad Complutense de Madrid, 28040 Madrid, Spain; 5 Instituto Pluridisciplinar, Universidad Complutense de Madrid, 28040 Madrid, Spain; 6 Instituto de Catálisis y Petroleoquímica, CSIC, Cantoblanco, 28049 Madrid, Spain

Correction to: *Scientific Reports* https://doi.org/10.1038/s41598-020-68085-0, published online 08 July 2020

The Supplementary Information file published with this Article contained an error in Supplementary Table S1 where the y value “0.7868(8)” for the Crystallographic data at I2 was incorrectly given as “0.7838(8)”.

The original Supplementary Information file is provided below.

This error has now been corrected in the Supplementary Information file that accompanies the original Article.

## Supplementary Information

Supplementary Information.

