# Supplementary material for: Author Correction: Enhanced stability in CH3NH3PbI3 hybrid perovskite from mechano-chemical synthesis: structural, microstructural and optoelectronic characterization
Source: Sci Rep. 2022 May 19;12:8416. doi: 10.1038/s41598-022-12534-5 (PMC9120503; doi:10.1038/s41598-022-12534-5)
Supplement: Supplementary file 1 — Supplementary Information. [file 41598_2022_12534_MOESM1_ESM.docx]

Enhanced stability in CH_3_NH_3_PbI_3_ hybrid perovskite from mechano-chemical synthesis: structural, microstructural and optoelectronic characterization.

Carlos A. López,*^1,2^ C. Abia,^1,3^ J.E. Rodrigues,^1^ F. Serrano-Sánchez,^1^ N.M. Nemes,^1,4^ J.L. Martínez,^1^ M.T. Fernandez-Díaz,^3^ N. Biškup,^5^ Consuelo Alvarez-Galván,^6^ Felix Carrascoso,^1^ Andres Castellanos-Gomez,^1^ and José A. Alonso*^1^

*^1^Instituto de Ciencia de Materiales de Madrid, CSIC, Cantoblanco 28049 Madrid, Spain.*

*^2^ Instituto de Investigaciones en Tecnología Química (INTEQUI), UNSL, CONICET and Facultad de Química, Bioquímica y Farmacia, UNSL, San Luis, 5700, Argentina.*

*^3^Institut Laue Langevin, BP 156X, F-38042 Grenoble, France.*

*^4^Departamento de Física de Materiales, Universidad Complutense de Madrid, E-28040 Madrid, Spain.*

*^5^Instituto Pluridisciplinar, Universidad Complutense de Madrid, E-28040 Madrid, Spain*

*^6^Instituto de Catálisis y Petroleoquímica, CSIC, Cantoblanco 28049 Madrid, Spain.*

[*ja.alonso@icmm.csic.es](mailto:*ja.alonso@icmm.csic.es); [calopez@unsl.edu.ar](mailto:caclopez@gmail.com)

***Figure S1.*** *Observed (crosses) calculated (black line) and difference (blue line) profiles after the Rietveld refinement from NPD data at (a) 180 K and (b) 140 K, corresponding to tetragonal (I*4*/mcm) and orthorhombic (Pnma) symmetry, respectively.*

***Figure S2.*** *Comparison of the unit-cell volume/Z as a function of temperature between the present sample and previous results from solvent-induced crystallization methods.*

***Table S1:*** *Crystallographic data for MAPbI_3_ phase in tetragonal system (I*4*/mcm) from NPD at 298 K.*

*a = 8.8556(3) Å, c = 12.6426(5) Å and V = 991.45(6) Å^3^*

|  | ***x*** | ***y*** | | ***z*** | | ***U_iso_^*^/U_eq_*** | ***f_occ_*** |
| --- | --- | --- | --- | --- | --- | --- | --- |
| **Pb1** | 0 | 0 | | 0 | | 0.0412(3) | 1 |
| **I1** | 0 | 0 | | 0.25 | | 0.058(2) | 1.00(4) |
| **I2** | 0.2868(8) | 0.7838(8) | | 0 | | 0.091(1) | 1.00(2) |
| **N1**  **C1** | 0.0774(2) | 0.4226(2) | | 0.2711(2) | | 0.038^*^ | 0.125(2)  0.125(2) |
| **N2**  **C2** | -0.03266(9) | 0.53266(9) | | 0.2198(1) | | 0.038^*^ | 0.125(2)  0.125(2) |
| **H11** | 0.1799(5) | 0.4678(5) | | 0.2880(4) | | 0.050^*^ | 0.125 |
| **H12** | 0.0915(3) | 0.3310(3) | | 0.2240(4) | | 0.050^*^ | 0.125 |
| **H13** | 0.0508(4) | 0.3789(4) | | 0.3399(3) | | 0.050^*^ | 0.125 |
| **H21** | -0.1265(5) | 0.4793(5) | | 0.2002(5) | | 0.050^*^ | 0.125 |
| **H22** | -0.0591(5) | 0.6205(5) | | 0.2652(7) | | 0.050^*^ | 0.125 |
| **H23** | 0.0132(4) | 0.5725(4) | | 0.1507(3) | | 0.050^*^ | 0.125 |
| R_p_ = 0.51%, R_wp_ = 0.69%, χ^2^ = 10.8, R_Bragg_ = 6.26% | | | | | | | |
| [Atomic displacement parameters (Å^2^)](file:///C:\Users\Usuario\AppData\Local\Temp\MAPbI3%20_atom_site_aniso_label) | | | | | | | |
|  | ***U^11^*** | ***U^22^*** | ***U^33^*** | | ***U^12^*** | ***U^13^*** | ***U^23^*** |
| **Pb1** | 0.0458(3) | 0.0458(3) | 0.0321(4) | | 0 | 0 | 0 |
| **I1** | 0.079(1) | 0.079(1) | 0.016(3) | | 0 | 0 | 0 |
| **I2** | 0.0852(7) | 0.0852(7) | 0.1010(8) | | 0.0773(7) | 0 | 0 |

***Table S2:*** *Crystallographic data for MAPbI_3_ phase in tetragonal system (I*4*/mcm) from NPD at 180 K.*

*a = 8.7810(2) Å, c = 12.6546(4) Å and V = 975.75(5) Å^3^*

|  | ***x*** | ***y*** | | ***z*** | | ***U_iso_^*^/U_eq_*** | ***f_occ_*** |
| --- | --- | --- | --- | --- | --- | --- | --- |
| **Pb1** | 0 | 0 | | 0 | | 0.0236(3) | 1 |
| **I1** | 0 | 0 | | 0.25 | | 0.027(1) | 1 |
| **I2** | 0.3012(5) | 0.8012(5) | | 0 | | 0.0427(4) | 1 |
| **N1**  **C1** | 0.0782(2) | 0.4218(2) | | 0.2870(2) | | 0.031^*^ | 0.230(2)  0.020(2) |
| **N2**  **C2** | -0.0314(1) | 0.5314(1) | | 0.2392(2) | | 0.031^*^ | 0.020(2)  0.230(2) |
| **H11** | 0.1833(4) | 0.4644(4) | | 0.3006(4) | | 0.050^*^ | 0.125 |
| **H12** | 0.0925(3) | 0.3300(3) | | 0.2403(4) | | 0.050^*^ | 0.125 |
| **H13** | 0.0449(3) | 0.3848(3) | | 0.3577(3) | | 0.050^*^ | 0.125 |
| **H21** | -0.1290(4) | 0.4818(4) | | 0.2166(5) | | 0.050^*^ | 0.125 |
| **H22** | -0.0636(5) | 0.6250(5) | | 0.2874(6) | | 0.050^*^ | 0.125 |
| **H23** | 0.0037(3) | 0.5819(3) | | 0.1680(3) | | 0.050^*^ | 0.125 |
| R_p_ = 0.52%, R_wp_ = 0.71%, χ^2^ = 11.1, R_Bragg_ = 4.82% | | | | | | | |
| [Atomic displacement parameters (Å^2^)](file:///C:\Users\Usuario\AppData\Local\Temp\MAPbI3%20_atom_site_aniso_label) | | | | | | | |
|  | ***U^11^*** | ***U^22^*** | ***U^33^*** | | ***U^12^*** | ***U^13^*** | ***U^23^*** |
| **Pb1** | 0.0230(2) | 0.0230(2) | 0.0248(4) | | 0 | 0 | 0 |
| **I1** | 0.0360(9) | 0.0360(9) | 0.008(2) | | 0 | 0 | 0 |
| **I2** | 0.0329(3) | 0.0329(3) | 0.0621(6) | | 0.0251(3) | 0 | 0 |

**Table S3:** Crystallographic data for MAPbI_3_ phase in orthorhombic system (*Pnma*) from NPD at 140 K.

*a* = 8.8451(4) Å, *b* = 12.5928(5) Å, *c* = 8.5834(4) Å and V = 956.05(8) Å^3^

|  | ***x*** | ***y*** | ***z*** | ***U_iso_*** | ***f_occ_*** |
| --- | --- | --- | --- | --- | --- |
| **Pb1** | 0 | 0 | 0.5 | 0.024(1) | 1 |
| **I1** | 1.0046(2) | 0.25 | 0.4473(1) | 0.029(1) | 1 |
| **I2** | 0.3094(1) | 0.0182(1) | 0.6871(1) | 0.029(1) | 1 |
| **N1** | 0.4360(1) | 0.25 | 0.4690(1) | 0.038 | 1 |
| **C2** | 0.5775(2) | 0.25 | 0.5528(2) | 0.038 | 1 |
| **H11** | 0.4507(6) | 0.25 | 0.3536(4) | 0.050 | 0.757(2) |
| **H12** | 0.3667(3) | 0.1839(1) | 0.4921(3) | 0.050 | 0.757(1) |
| **H21** | 0.5547(6) | 0.25 | 0.6682(4) | 0.050 | 0.757(2) |
| **H22** | 0.6451(4) | 0.1839(1) | 0.5324(3) | 0.050 | 0.757(1) |
| **H13** | 0.352(1) | 0.25 | 0.544(1) | 0.050 | 0.243(2) |
| **H14** | 0.423(1) | 0.1839(1) | 0.4041(7) | 0.050 | 0.243(1) |
| **H23** | 0.662(1) | 0.25 | 0.477(1) | 0.050 | 0.243(2) |
| **H24** | 0.590(1) | 0.1839(1) | 0.6178(7) | 0.050 | 0.243(1) |
| R_p_ = 0.60%, R_wp_ = 0.82%, χ^2^ = 14.6, R_Bragg_ = 8.82% | | | | | |

**Table S4:** Crystallographic data for MAPbI_3_ phase in orthorhombic system (*Pnma*) from NPD at 100 K.

*a* = 8.83213(7) Å, *b* = 12.5812(1) Å, *c* = 8.56607(8) Å and V = 951.85(1) Å^3^

|  | ***x*** | ***y*** | ***z*** | ***U_iso_*** | ***f_occ_*** |
| --- | --- | --- | --- | --- | --- |
| **Pb1** | 0 | 0 | 0.5 | 0.017(1) | 1 |
| **I1** | 1.0106(2) | 0.25 | 0.4445(1) | 0.029(1) | 1 |
| **I2** | 0.31116(9) | 0.01794(8) | 0.68598(8) | 0.029(1) | 1 |
| **N1** | 0.4370(1) | 0.25 | 0.4671(1) | 0.038 | 1 |
| **C2** | 0.5779(2) | 0.25 | 0.5545(2) | 0.038 | 1 |
| **H11** | 0.4544(5) | 0.25 | 0.3518(3) | 0.050 | 0.831(2) |
| **H12** | 0.3677(3) | 0.18353(6) | 0.4888(3) | 0.050 | 0.831(1) |
| **H21** | 0.5553(5) | 0.25 | 0.6706(3) | 0.050 | 0.831(2) |
| **H22** | 0.6466(3) | 0.18353(6) | 0.5332(3) | 0.050 | 0.831(1) |
| **H13** | 0.351(1) | 0.25 | 0.540(2) | 0.050 | 0.169(2) |
| **H14** | 0.428(2) | 0.18353(6) | 0.4016(8) | 0.050 | 0.169(1) |
| **H23** | 0.663(1) | 0.25 | 0.481(2) | 0.050 | 0.169(2) |
| **H24** | 0.587(2) | 0.18353(6) | 0.6200(8) | 0.050 | 0.169(1) |
| R_p_ = 0.59%, R_wp_ = 0.81%, χ^2^ = 14.2, R_Bragg_ = 7.99% | | | | | |


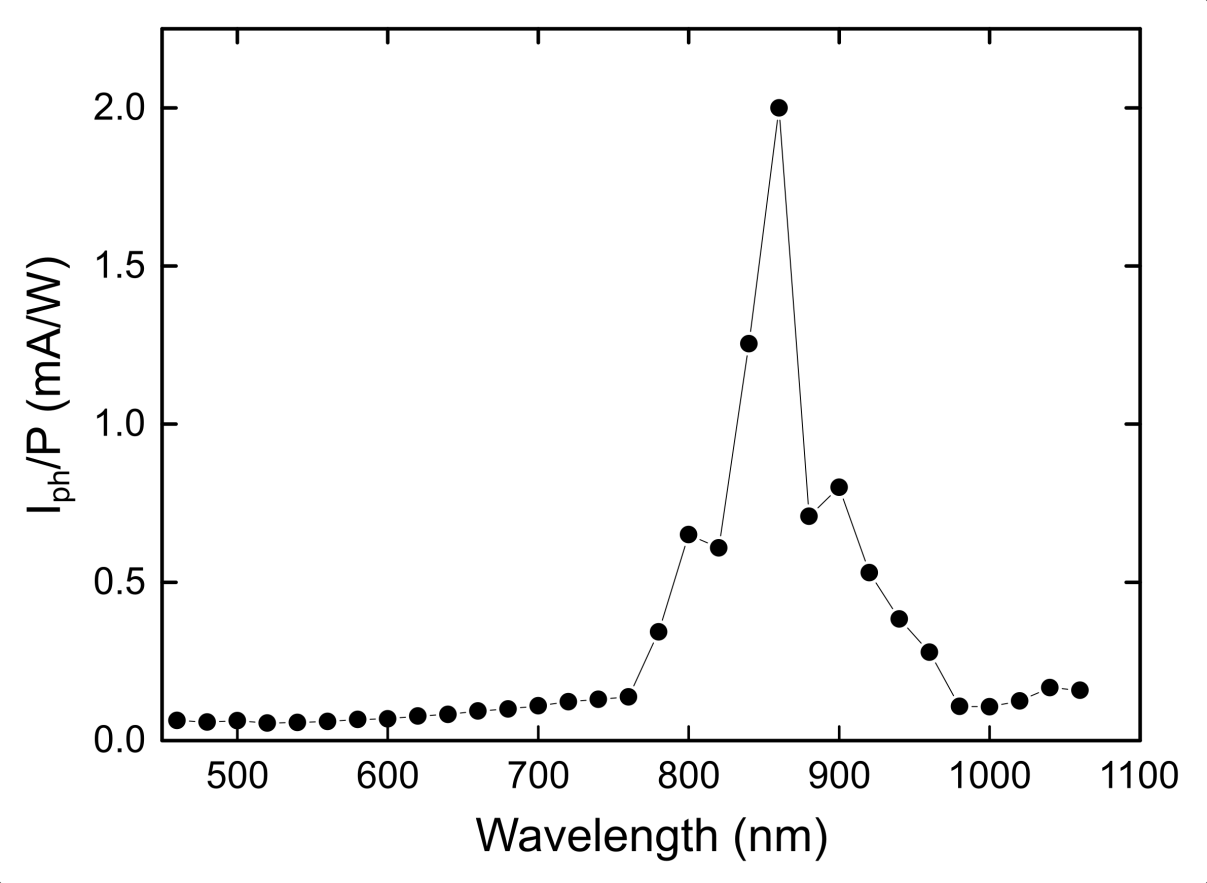


***Figure S3****. Shape of the spectrum in an Iph/power vs wavelength representation. Dividing the photocurrent by the power density reaching de device would provide a conceptually correct responsivity spectrum only if the power density is kept constant for all wavelengths.*
